# Evaluation of surgical revascularization procedure outcomes for adult Moyamoya disease: a computed tomography perfusion-based study

**DOI:** 10.1186/s13244-023-01519-1

**Published:** 2023-11-04

**Authors:** Xuexia Yuan, Hao Yu, Zhanguo Sun, Jiaxing Wu, Lingyun Gao, Zhen Chong, Feng Jin, Yueqin Chen, Deguo Liu

**Affiliations:** 1 Department of Radiology, Affiliated Hospital of Jining Medical University, Jining, China; 2 Siemens Healthineers, No. 399, West Haiyang Road, Shanghai, China; 3 Department of Neurosurgery, Affiliated Hospital of Jining Medical University, Jining, China

**Keywords:** Moyamoya disease, Perfusion imaging, Revascularization, Collaterals

## Abstract

**Background:**

The effectiveness of surgical interventions, whether direct or indirect, for Moyamoya disease (MMD) remains controversial. This study aims to investigate CT perfusion (CTP) as an objective method to evaluate the outcomes of different surgical modalities for adult MMD.

**Methods:**

The clinical and imaging data of 41 patients who underwent superficial temporal artery-middle cerebral artery (STA-MCA) bypass and 43 who received encephaloduroarteriosynangiosis (EDAS) were retrospectively analyzed. Intra- and intergroup differences in the Modified Rankin Scale (mRS) score, the change in clinical symptoms, collateral grade, and CTP parameters pre- and postoperatively were compared.

**Results:**

The overall level of the change in clinical symptoms in the STA-MCA group was higher than in the EDAS group (*p* < 0.05). In the operative area, the relative cerebral blood flow (rCBF) was significantly higher whereas the relative time to peak (rTTP) and the relative mean transit time (rMTT) were significantly lower in the STA-MCA and EDAS groups postoperatively than preoperatively (all *p* < 0.05). In the ipsilateral frontal lobe and basal ganglia, the postoperative rCBF was significantly higher, and the rTTP was significantly lower than the preoperative in the STA-MCA group (all *p* < 0.05). The postoperative rCBF improvement was higher in each brain area for STA-MCA than in the EDAS group (all *p* < 0.05).

**Conclusion:**

Highlighting the utility of CTP, this study demonstrates its effectiveness in assessing postoperative cerebral hemodynamic changes in adult MMD patients. STA-MCA yielded a larger postoperative perfusion area and greater improvement compared to EDAS, suggesting CTP’s potential to elucidate symptom variation between two surgical revascularization procedures.

**Critical relevance statement:**

We analyzed computed tomography perfusion parameters in pre- and postoperative adult Moyamoya disease patients undergoing superficial temporal artery-middle cerebral artery bypass and encephaloduroarteriosynangiosis. Our findings suggest computed tomography perfusion’s potential in objectively elucidating symptom variations between these surgical revascularization approaches for MMD.

**Key points:**

• Postoperative perfusion improvement is only confined to the operative area after EDAS.

• Besides the operative area, postoperative perfusion in the ipsilateral frontal lobe and basal ganglia was also improved after STA-MCA.

• The degree of perfusion improvement in each brain area in the STA-MCA group was generally greater than that in the EDAS group.

**Graphical Abstract:**

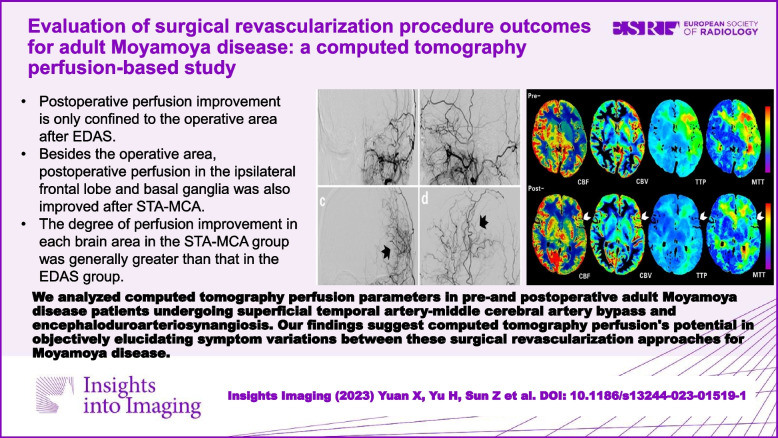

## Background

Moyamoya disease (MMD) is a cerebrovascular condition with unknown etiology characterized by progressive stenosis or occlusion at the ends of bilateral internal carotid arteries with secondary formation of an abnormal vascular network at the base of the brain [1, 2]. Surgical revascularization is the preferred treatment method, which includes direct revascularization, indirect revascularization, or a combination of both [3, 4]. The ideal treatment should be effective in improving symptoms and preventing recurrent stroke. The superficial temporal artery-middle cerebral artery (STA-MCA) anastomosis and encephaloduroarteriosynangiosis (EDAS) are commonly used in direct and indirect procedures, respectively. Different medical institutions have reported the safety and effectiveness of these surgical procedures for MMD, with favorable clinical outcomes [5–10]. However, there is ongoing debate regarding the optimal method of revascularization for MMD [7–15].

Imaging examination may potentially provide objective evidence for the evaluation of postoperative MMD outcomes [5]. Postoperative collateral grade by digital subtraction angiography (DSA) is the main method for evaluating surgical procedure outcomes for MMD; however, DSA does not fully reflect the cerebral hemodynamic status [16]. Brain perfusion imaging techniques, such as single-photon emission computed tomography (SPECT) and magnetic resonance perfusion, are valuable for evaluating cerebral perfusion in MMD patients. However, these methods can be limited by artifacts [17]. Computed tomography perfusion (CTP) is a commonly used method for clinical evaluation and for quantitative evaluation of subtle changes with multiple parameters. Previous studies of postoperative cerebral hemodynamic changes in patients with MMD mostly focused on the operative area [5, 18] and rarely investigated other regions of the hemisphere ipsilateral; however, perfusion changes in these regions also affected the changes in clinical symptoms [19–21].

This study retrospectively analyzed the clinical and imaging data of adult patients with MMD who received STA-MCA anastomosis or EDAS to objectively evaluate outcomes of different surgical procedures for the treatment of MMD using CTP. This study aimed to investigate whether quantitative data based on hemodynamics from CTP could serve as an objective basis for explaining the efficacy of different surgical procedures.

## Methods

### Study participants

**Fig. 1:**
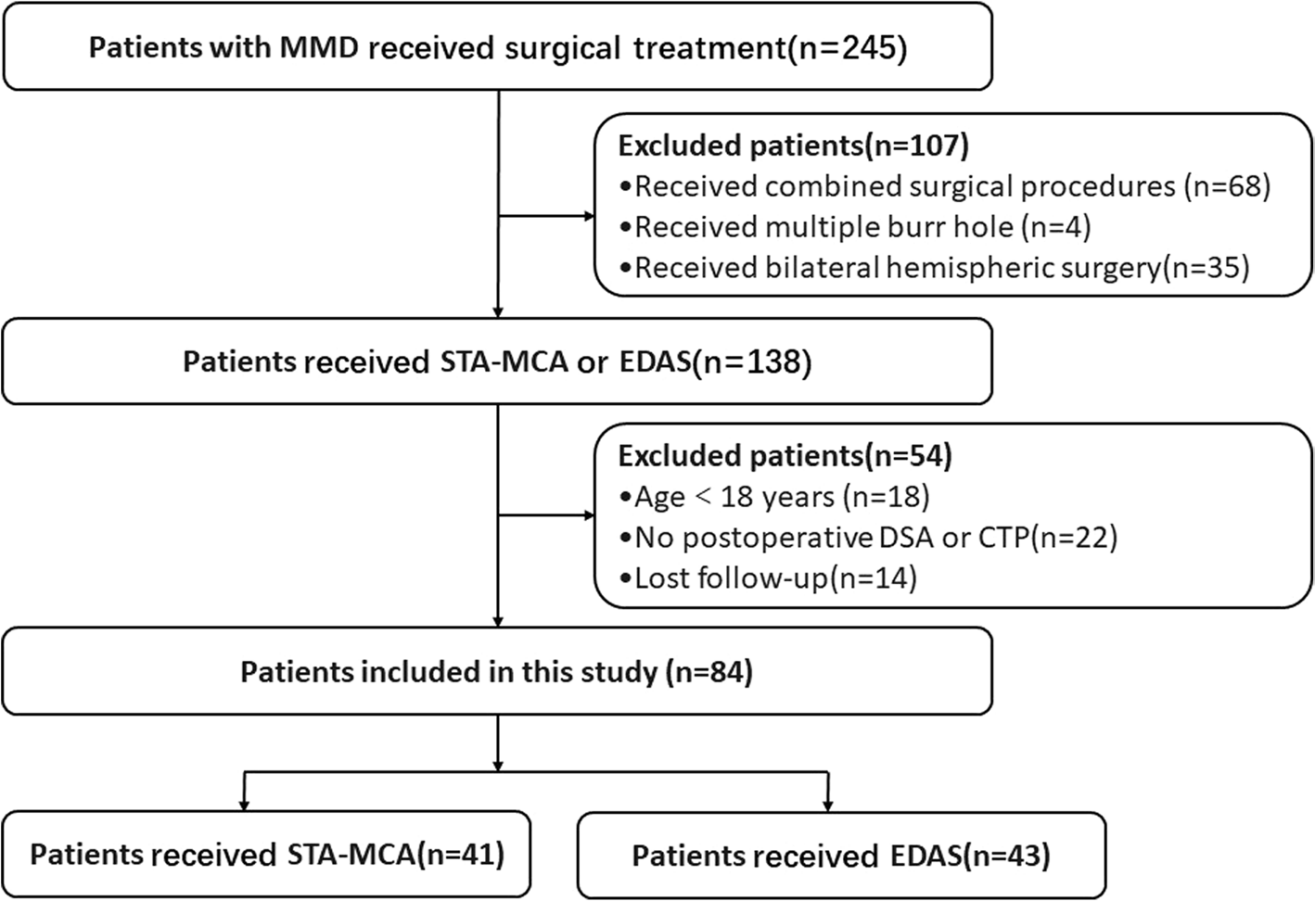
Flowchart of the study. MMD, Moyamoya disease; STA-MCA, superficial temporal artery-middle cerebral artery; DSA, digital subtraction angiography; EDAS, encephaloduroarteriosynangiosis; CTP, computed tomography perfusion

This study was approved by the institutional medical ethics committee, and informed consent was waived. Data of 245 consecutive patients with MMD who underwent STA-MCA anastomosis or EDAS between April 2017 and August 2022 were retrospectively analyzed. The diagnostic criteria for MMD were based on the guidelines published in 2022 [2]. The inclusion criteria of patients were as follows: (1) age ≥ 18 years, (2) complete pre- and postoperative clinical data, (3) complete pre- and postoperative CTP and DSA examinations, and (4) follow-up at least 6 months after surgery. Patients with MMD who underwent combined surgical procedures or bilateral hemispheric surgery were excluded. Eighty-four patients were enrolled (Fig. 1). During the follow-up period, none of the patients in either group has experienced a new ischemic or hemorrhagic event.

### Treatment methods

Surgeries were performed by an experienced neurosurgical expert (F.J. with 25 years of experience in neurosurgery). STA-MCA anastomosis refers to the end-to-side anastomosis of the dissected frontal branch or apical branch of the superficial temporal artery with the M4 MCA segment [9]. EDAS refers to the dissected frontal branch or apical branch adhesions in the superficial temporal artery to the arachnoid mater, with its edge sutured to the dura mater [22].

The principles of the surgical strategies at our institution were as follows. First, the selection of surgical hemispheres is mainly based on clinical manifestation, and symptomatic hemisphere was the preferred side for revascularization surgery. Second, for patients without an obvious symptomatic hemisphere, CT perfusion shows that the hemisphere with more severe hypoperfusion injury is the preferred treatment hemisphere. Third, STA-MCA was the favored surgical modality for most patients, whether in ischemic or hemorrhagic patients. An EDAS was performed only when the donor or recipient artery was too small or fragile to perform artery anastomosis.

### Imaging acquisition and evaluation

**Fig. 2:**
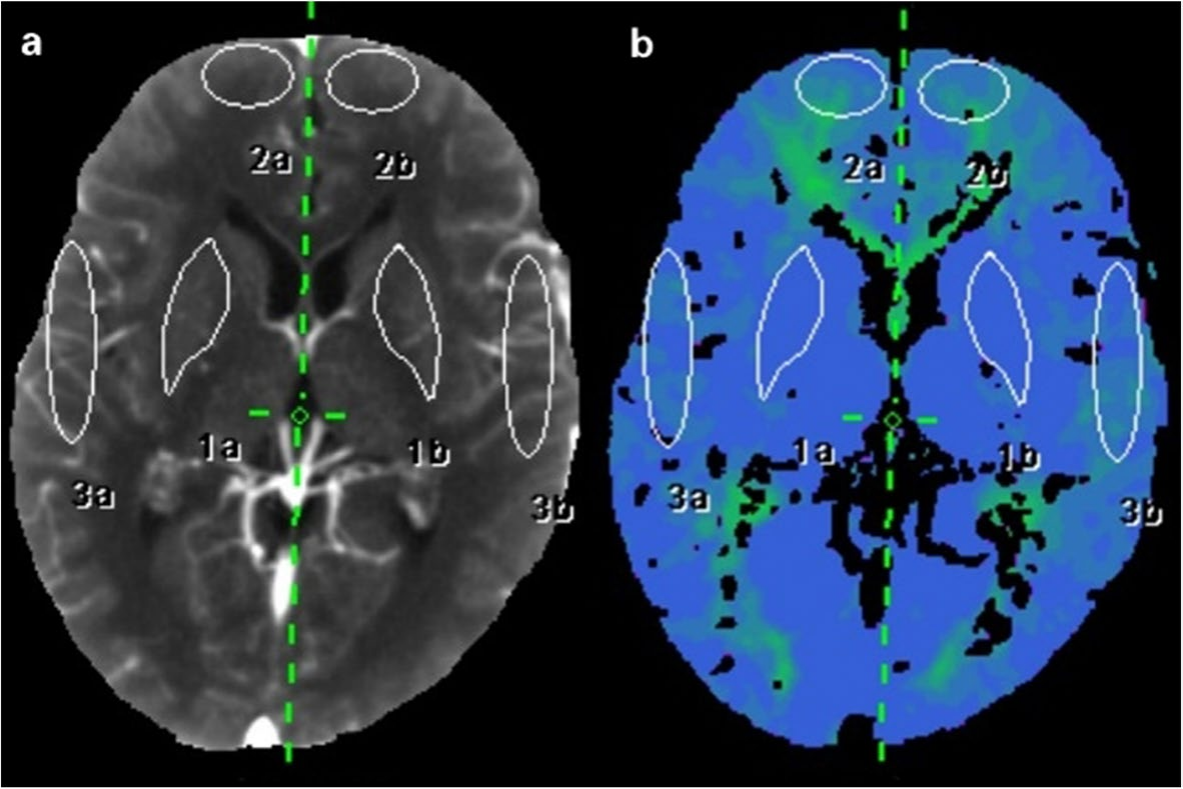
Example of regions of interest (ROIs) of the frontal lobe, operative area, and basal ganglia, drawn in the reference CT image (**a**) and time-to-peak map (**b**)

All CTPs were performed on a 128-slice CT scanner (SOMATOM Definition Flash, Siemens Healthineers) using a MEDRAD high-pressure syringe. After a non-contrast brain scanning, 50 mL of iopromide (Ultravist 300 or 370; Bayer Schering) was injected for multiphase dynamic scanning. Reconstructed CTP images were transferred to the multimodality postprocessing station (Syngo MMWP, VE 40C, Siemens Healthcare) using the VPCT perfusion software to obtain various CTP parameters: cerebral blood flow (CBF), cerebral blood volume (CBV), time to peak (TTP), and mean transit time (MTT). These images were independently analyzed by 2 neuroradiologists (D.L. and Y.C. with 18 and 22 years of experience in neuroradiology, respectively) who were blinded to the clinical data, type of surgery, and DSA results, and the CTP interpretation training program was performed for both to ensure the consistency of interpreting the standard. The first artery to reach peak enhancement on the time-attenuation curve was selected as the arterial input function. The placement of the regions of interest (ROIs) should avoid the large blood vessels, artifacts, hemorrhage, and infarct area. In addition to the operative area, ROIs of the frontal lobes and basal ganglia, representing the blood supply area of the anterior cerebral artery and deep brain tissue, were also conducted, respectively [23–25] (Fig. 2). In our study, the relative CTP values were defined as absolute CTP values in the surgical side divided by absolute CTP values in the pons [26]. The change in postoperative perfusion was defined as differences between relative parameters pre- and postoperatively.

DSA images were acquired using transfemoral cerebral angiography, such as superselective angiography of bilateral external carotid arteries. The Matsushima grading system [27] was used to classify the extent of collateralization: grade 0 refers to no evident collateralization, and grades 1, 2, and 3 are defined as < 1/3, between 1/3 and 2/3, and > 2/3 of the MCA territory perfused by collateralization, respectively.

### Clinical outcome assessment

Clinical outcomes were evaluated by two neurosurgical experts (G.S. and H.Z. with 22 and 20 years of experience in neurosurgery, respectively), both blinded to the result of imaging and details of surgery, through the patient files. Any inconsistency was resolved by senior supervisors’ consultation and thorough discussion on roundtable conference with the Academic Committee. Refer to previous reports [28–30], the change in clinical symptoms was classified into four levels: excellent, preoperative symptoms (such as transient ischemic attacks or seizures) had totally disappeared without fixed neurological deficits; good, symptoms had totally disappeared, but neurological deficits remained; fair, symptoms persisted, albeit less frequently; and poor, symptoms remained unchanged or worsened. “Excellent” and “good” were considered as favorable remission of clinical symptoms. Moreover, neurological status was evaluated using the Modified Rankin Scale (mRS) score either.

### Statistical analysis

Intra- and inter-observer correlations for the CBF values were analyzed by estimating the ICCs (the degree of agreement is interpreted as poor for ICC < 0.5, moderate for ICC 0.5–0.75, good for ICC 0.75–0.9, and excellent for ICC > 0.9) [31]. The Statistical Package for the Social Sciences 25.0 statistical software was used for data analysis. Normally distributed measurement data are expressed as mean ± standard deviation ($\overline{x }$ ± SD) and compared using the two-sample *t*-test and paired *t*-test. Non-normally distributed measurement data are expressed as the median (interquartile range [IQR]) and compared using Wilcoxon’s rank-sum test. Count data are expressed as number of patients (percentage) [*n* (%)] and compared using the chi-square (*χ*^2^) test or Fisher’s exact probability method. Grade data were compared using Wilcoxon’s rank-sum test. Spearman rank correlation analysis was used to evaluate the correlation between hemodynamic improvement and clinical symptom remission. *p* < 0.05 was considered statistically significant.

## Results

### Grouping and basic characteristics

The final cohort comprised 84 patients with a mean age of 45 years (22–61 years), including 42 males and 42 females. A total of 65 patients presented with ischemia and 19 patients with hemorrhage. Forty-one patients were included in the STA-MCA group, and 43 patients were included in the EDAS group. The basic characteristics of the enrolled patients, as well as those of the hemispheres, were separately summarized in Table 1. No significant differences were observed between the groups in terms of age, male-to-female ratio, clinical manifestations, surgical side, Suzuki stage, preoperative mRS scores, or postoperative follow-up duration (all *p* > 0.05).

**Table1:** Preoperative and postoperative demographics of patients with adult Moyamoya disease

Characteristic	STA-MCA (*n* = 41)	EDAS (*n* = 43)	Value	*p* value
**Age (mean ± SD), years**	44.7 ± 9.7	45.2 ± 8.6	0.240	0.811^a^
**Sex**			0.429	0.513^b^
Male	22 (53.7)	20 (46.5)		
Female	19 (46.3)	23 (53.5)		
**Preoperative clinical symptoms**			1.407	0.235^b^
Ischemia	34 (82.9)	31 (72.1)		
Hemorrhage	7 (17.1)	12 (27.9)		
**Surgical side**			0.444	0.505^b^
Lt	18 (43.9)	22 (51.2)		
Rt	23 (56.1)	21 (48.8)		
**Suzuki stage**			0.156	0.876^c^
II	4 (9.8)	5 (11.6)		
III	21 (51.2)	23 (53.5)		
IV	10 (24.4)	6 (14.0)		
V	6 (14.6)	9 (20.9)		
**Preoperative mRS score**	1 (1.2)	1 (1.2)	0.281	0.778^c^
**Clinical outcome**			2.128	0.033^c^
Poor	3 (7.3)	8 (18.6)		
Fair	5 (12.2)	10 (23.3)		
Good	9 (21.9)	8 (18.6)		
Excellent	24 (58.5)	17 (39.5)		
**Revascularization grade**			0.666	0.506^c^
Grade 0	5 (12.2)	8 (18.6)		
Grade 1	10 (24.4)	10 (23.3)		
Grade 2	18 (43.9)	18 (41.9)		
Grade 3	8 (19.5)	7 (16.3)		
**Postoperative mRS score**	0 (0.1)	0 (0.1)	1.193	0.233^c^
**Postoperative follow-up (months)**	6.4 ± 0.6	6.5 ± 0.7	0.873	0.385^a^

*STA-MCA* superficial temporal artery-middle cerebral artery, *EDAS* encephaloduroarteriosynangiosis, *mRS* Modified Rankin Scale

^a^*t*-test

^b^*χ*^2^ test

^c^Wilcoxon’s rank-sum test

### Comparison of the clinical outcome between the EDAS and STA-MCA groups

The change in clinical symptoms in 84 patients postoperative was classified as follows: “excellent” in 41 (48.81%) patients, “good” in 17 (20.24%), “fair” in 15 (17.86%), and “poor” in 11 (13.10%). The favorable remission rate was 69.05%. The overall level of the change in clinical symptoms in the STA-MCA group was significantly higher than that in the EDAS group (Table 1), and the favorable remission was also significantly higher than that in the EDAS group [33 (80.5%) vs. 25 (58.1%),* p* = 0.027].

The mRS scores of 84 patients were 1 (IQR 1–2) pre-operation and 0 (IQR 0–1) post-operation, with a significant difference (*z* = 6.053, *p* < 0.001). The postoperative mRS scores were significantly lower than that of preoperative in both STA-MCA [1 (IQR 1–2) pre-operation vs. 0 (IQR 0–1) post-operation, *z* = 4.624, *p* < 0.001] and EDAS [1 (IQR 1–2) pre-operation vs. 0 (IQR 0–1) post-operation, *z* = 3.938,* p* < 0.001), respectively] groups. There were no statistical differences in post-operative mRS scores between STA-MCA and EDAS groups (*z* = 1.193,* p* = 0.233) (Table 1).

### Comparison of the DSA-evaluated collateralization grade between the EDAS and STA-MCA groups

**Fig. 3:**
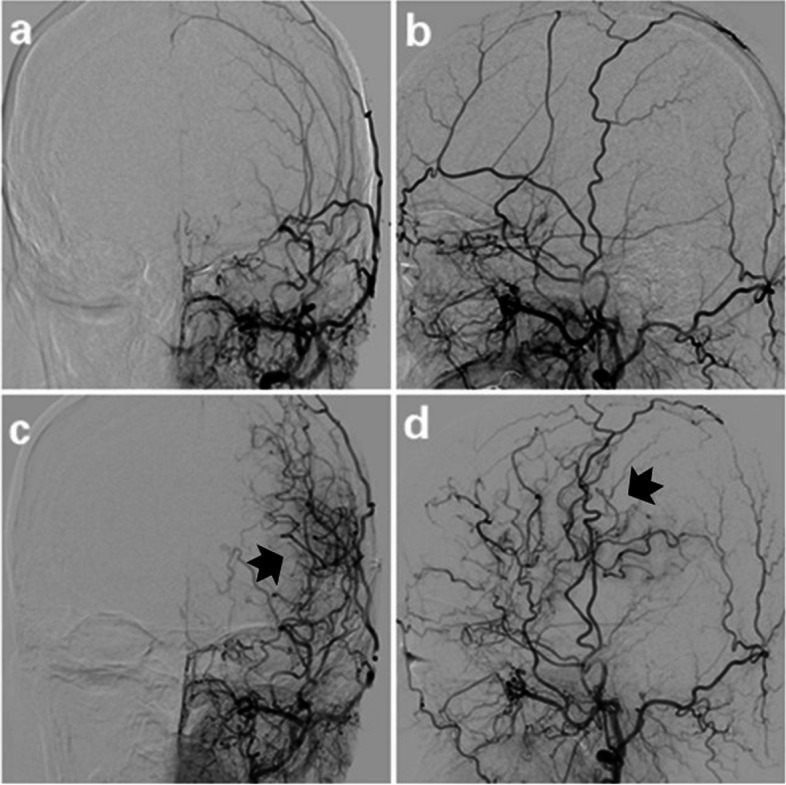
Superselective angiography of the left external carotid artery images of a 45-year-old female patient with MMD diagnosed by left internal carotid artery occlusion. **a**, **b** The left superficial temporal artery trunk and branch were clearly visible preoperatively. **c**, **d** At 9 months after STA-MCA, the branches of the left superficial temporal artery flow extended into the cranium with patent graft and grade 3 collateralization formation (black arrows)

**Fig. 4:**
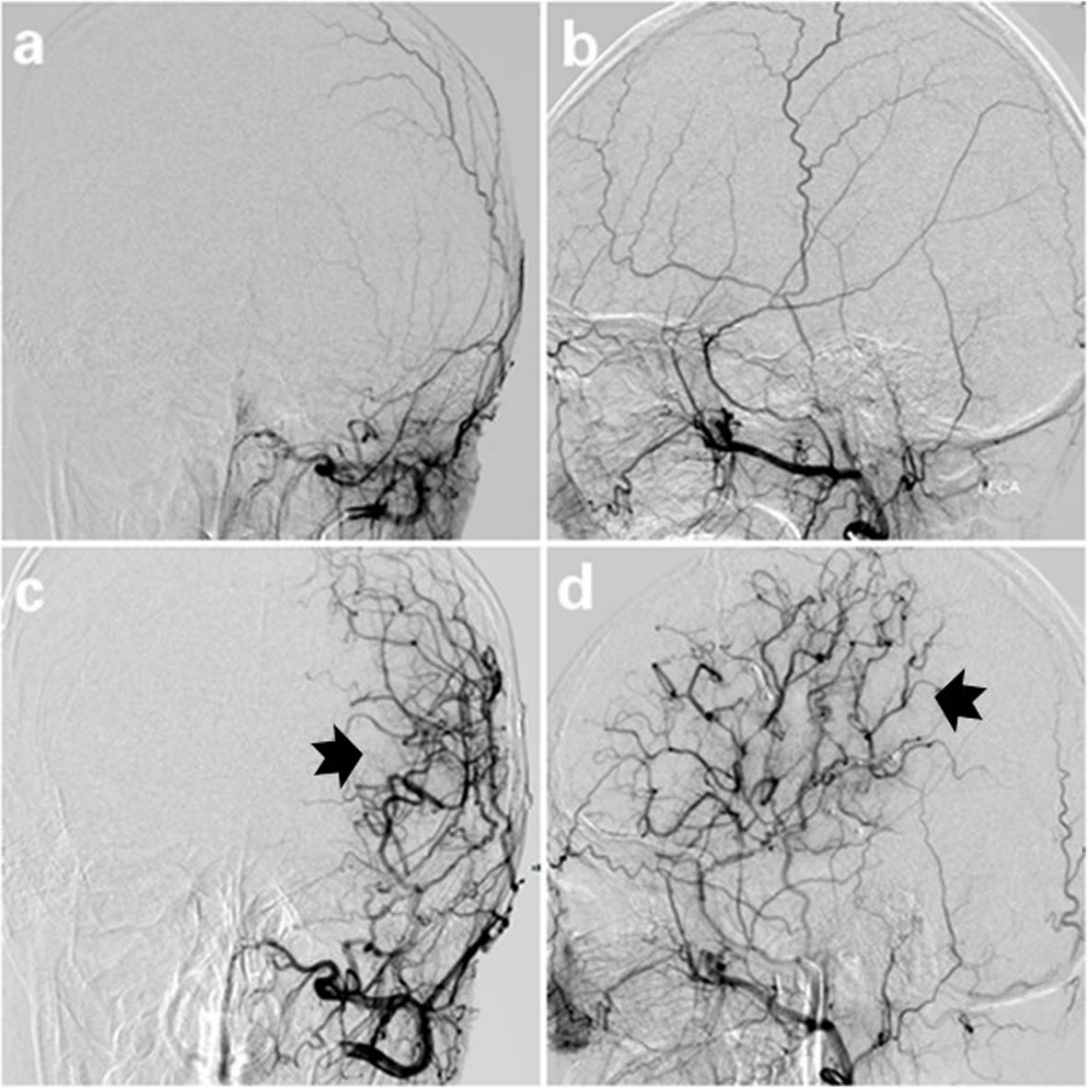
Superselective angiography of the left external carotid artery images of a 22-year-old female patient with MMD diagnosed by left internal carotid artery occlusion. **a**, **b** The left superficial temporal artery trunk and branch were clearly visible preoperatively. **c**, **d** At 8 months after EDAS, the branches of the left superficial temporal artery flow extending into the cranium with patent graft and grade 3 collateralization formation (black arrows)

Postoperative DSA demonstrated that neovascularization occurred in 71 of 84 (84.52%) surgical hemispheres (Figs. 3 and 4) but not found in 13 (15.48%) hemispheres. The grade of postoperative collateralization did not significantly differ between the STA-MCA and EDAS groups (*z* = 0.666, *p* = 0.506) (Table 1).

### Intra‑ and inter‑observer agreement

The interclass correlation coefficients (95% CI), reflecting interobserver reproducibility for Pre-CBF and Post-CBF, demonstrated strong agreement ranging from 0.91 to 0.97, which suggests excellent reliability. Similarly, the intraclass correlation coefficients (95% CI) for intra-observer reproducibility exhibited robust consistency, ranging from 0.91 to 0.96 (Table 2).

**Table 2:** Intra- and inter-observer consistency evaluation of CBF parameters in various ROIs using ICC measurement

ICC		STA-MCA		EDAS	
		Intra (95% CI)	Inter (95% CI)	Intra (95% CI)	Inter (95% CI)
Operated area	Pre-CBF	0.96 (0.81–0.99)	0.95 (0.85–0.98)	0.95 (0.92–0.98)	0.96 (0.92–0.98)
	Post-CBF	0.96 (0.93–0.98)	0.93 (0.88–0.96)	0.95 (0.91–0.97)	0.94 (0.89–0.97)
Basal ganglia	Pre-CBF	0.95 (0.91–0.97)	0.96 (0.92–0.98)	0.94 (0.90–0.97)	0.95 (0.91–0.97)
	Post-CBF	0.94 (0.89–0.97)	0.92 (0.86–0.96)	0.91 (0.83–0.95)	0.91 (0.84–0.95)
Frontal lobe	Pre-CBF	0.95 (0.91–0.97)	0.93 (0.87–0.96)	0.95 (0.92–0.98)	0.96 (0.93–0.98)
	Post-CBF	0.93 (0.88–0.96)	0.93 (0.86–0.96)	0.92 (0.85–0.96)	0.94 (0.88–0.97)
Pons	Pre-CBF	0.95 (0.91–0.98)	0.97 (0.82–0.99)	0.94 (0.89–0.97)	0.91 (0.83–0.95)
	Post-CBF	0.91 (0.83–0.95)	0.93 (0.87–0.96)	0.92 (0.85–0.95)	0.91 (0.84–0.95)

*ICC* intra-class correlation coefficient, *CBF*cerebral blood flow, *STA-MCA* superficial temporal artery-middle cerebral artery, *EDAS* encephaloduroarteriosynangiosis, *Pre-* preoperative, *Post-* postoperative

### Comparison of the pre- and postoperative CTP parameters of all patients

**Fig. 5:**
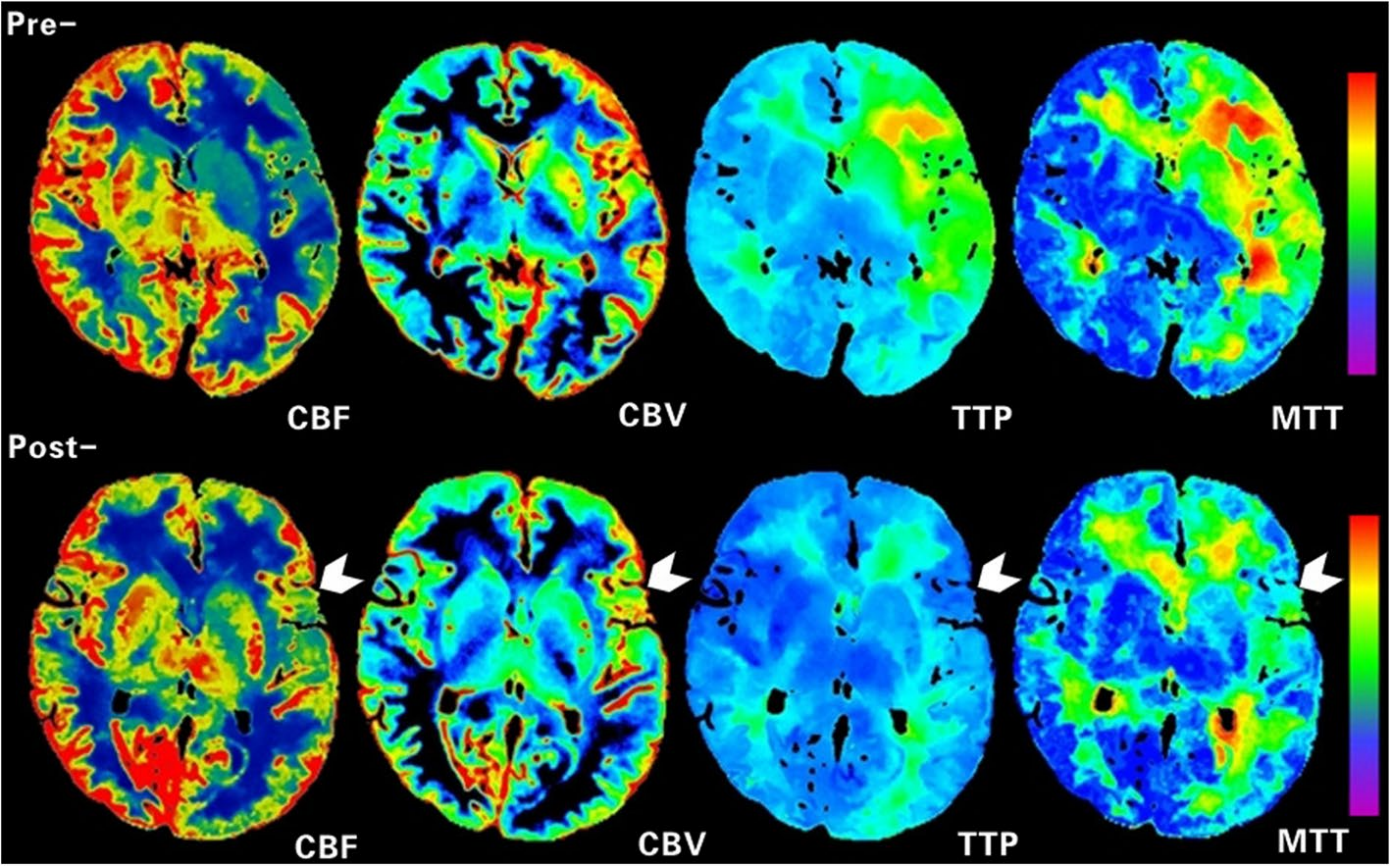
CTP images before (top) and after (bottom) STA-MCA of the same patient as in Fig. 3. Preoperatively, CBF was significantly low, CBV was slightly high, and TTP and MTT were significantly delayed in the left hemisphere. Postoperatively, cerebral perfusion in the left frontoparietal lobe and basal ganglia was improved, as manifested by increased CBF, similar CBV, and shortened TTP and MTT

**Fig. 6:**
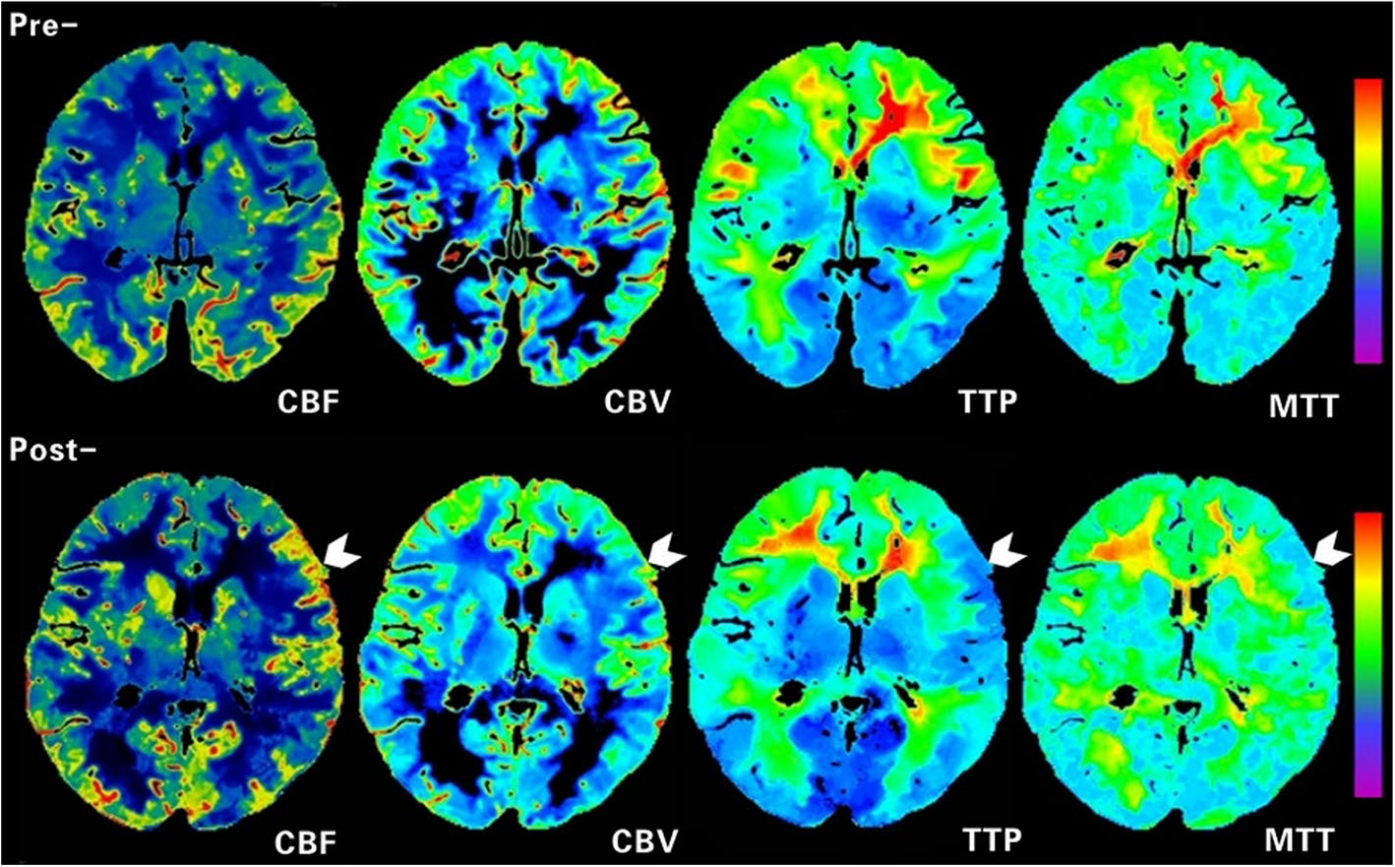
CTP images before (top) and after (bottom) EDAS of the same patient as in Fig. 4. Preoperatively, CBF was significantly low, CBV was slightly low, and TTP and MTT were significantly delayed in the left hemisphere. Postoperatively, cerebral perfusion in the left frontoparietal lobe was improved, as manifested by increased CBF, similar CBV, and shortened TTP and MTT

The postoperative rCBF was significantly higher, and rCBV, rTTP, and rMTT were significantly lower than the preoperative (all *p* < 0.05) in the operative area (Table 3). The perfusion of 78 (78/84, 92.9%) patients showed improvement after surgery. The postoperative hemodynamic improvement was correlated with the change in clinical symptoms (*r* = 0.714, *p* < 0.05). Figures 5 and 6 show the CTP images of a typical surgical hemisphere.

**Table 3:** The relative preoperative and postoperative CTP parameters of 84 patients

Characteristic	Preoperative	Postoperative	*t* value	*p* value
rCBF	1.03 ± 0.35	1.29 ± 0.33	5.844	< 0.001
rCBV	1.49 ± 0.42	1.37 ± 0.36	2.322	0.023
rTTP	1.22 ± 0.19	1.07 ± 0.10	7.270	< 0.001
rMTT	1.70 ± 0.64	1.20 ± 0.43	6.598	< 0.001

*rCBF* relative cerebral blood flow, *rCBV* relative cerebral blood volume, *rTTP* relative time to peak, *rMTT* relative mean transit time

### Comparison of the pre- and postoperative CTP parameters intra EDAS and STA-MCA groups

The results of comparison pre- and postoperative CTP parameters intra EDAS and STA-MCA groups are summarized in Table 4. In the operative area, the postoperative rCBF was significantly higher and the rTTP and rMTT were significantly lower than the preoperative in both the EDAS and STA-MCA groups (all *p* < 0.05). The postoperative rCBV was significantly lower than the preoperative in the EDAS group (*p* < 0.05), but there was no significant difference in rCBV between pre- and postoperative in the STA-MCA group (*p* > 0.05). In the ipsilateral basal ganglia, the postoperative rCBF was significantly higher, and the rCBV, rTTP, and rMTT were significantly lower than the preoperative in the STA-MCA group (all *p* < 0.05); however, no significant differences in the CTP parameters were found in the EDAS group (all *p* > 0.05). Similarly, in the ipsilateral frontal lobe, the postoperative rCBF was significantly higher, and the rTTP was significantly lower than the preoperative in the STA-MCA group (all* p* < 0.05). However, there were no significant differences in the CTP parameters in the EDAS group (all* p* > 0.05).

**Table 4:** The relative preoperative and postoperative CTP parameters in patients

Variable		STA-MCA (*n* = 41)		*t* value	*p* value	EDAS (*n* = 43)		*t* value	*p* value
		Preoperative	Postoperative			Preoperative	Postoperative		
Operated area	rCBF	1.01 ± 0.34	1.37 ± 0.28	6.102	< 0.001	1.04 ± 0.36	1.20 ± 0.35	2.594	0.013
	rCBV	1.55 ± 0.44	1.42 ± 0.44	1.405	0.168	1.43 ± 0.39	1.32 ± 0.26	2.180	0.035
	rTTP	1.18 ± 0.14	1.03 ± 0.09	5.518	< 0.001	1.26 ± 0.22	1.10 ± 0.10	4.862	< 0.001
	rMTT	1.75 ± 0.62	1.11 ± 0.44	5.409	< 0.001	1.64 ± 0.68	1.29 ± 0.42	3.981	< 0.001
Basal ganglia	rCBF	1.21 ± 0.37	1.34 ± 0.35	2.276	0.028	1.40 ± 0.35	1.30 ± 0.30	1.413	0.165
	rCBV	1.36 ± 0.29	1.23 ± 0.36	2.101	0.042	1.53 ± 0.35	1.42 ± 0.34	1.725	0.092
	rTTP	1.04 ± 0.13	0.97 ± 0.14	2.899	0.006	1.11 ± 0.44	1.08 ± 0.14	0.488	0.628
	rMTT	1.22 ± 0.49	1.02 ± 0.44	2.572	0.038	1.14 ± 0.33	1.13 ± 0.33	0.158	0.875
Frontal lobe	rCBF	0.97 ± 0.28	1.10 ± 0.27	2.955	0.005	1.03 ± 0.42	0.98 ± 0.26	0.734	0.467
	rCBV	1.22 ± 0.30	1.10 ± 0.37	1.763	0.086	1.26 ± 0.40	1.24 ± 0.24	0.216	0.830
	rTTP	1.14 ± 0.15	1.09 ± 0.13	2.237	0.031	1.20 ± 0.31	1.17 ± 0.17	0.826	0.413
	rMTT	1.54 ± 0.73	1.36 ± 0.64	1.482	0.146	1.57 ± 0.67	1.53 ± 0.55	0.432	0.668

*rCBF* relative cerebral blood flow, *rCBV* relative cerebral blood volume, *rTTP* relative time to peak, *rMTT* relative mean transit time, *STA-MCA* superficial temporal artery-middle cerebral artery, *EDAS* encephaloduroarteriosynangiosis

### Comparison of the change of CTP parameters pre- and postoperative between the EDAS and STA-MCA groups

In the operative area, the change of rCBF in the STA-MCA group was significantly higher than that in the EDAS group, and the change of rMTT was significantly lower than that in the EDAS group (all *p* < 0.05). In the basal ganglia, the change of rCBF in the STA-MCA group was significantly higher than that in the EDAS group, and the changes of rTTP and rMTT were significantly lower than that in the EDAS group (all* p* < 0.05). In the frontal lobe, the change of rCBF in the STA-MCA group was significantly higher than that in the EDAS group (*p* < 0.05) (Table 5).

**Table 5:** Comparison of the change in postoperative perfusion between the two groups

Variable		postoperative perfusion change		Value	*p* value
		STA-MCA (*n* = 41)	EDAS (*n* = 43)		
Operated area	rCBF	0.36 ± 0.37	0.26 ± 0.41	2.240	0.028^a^
	rCBV	− 0.10 (− 0.52 to 0.22)	− 0.07 (− 0.28 to 0.17)	0.398	0.690^b^
	rTTP	− 0.16 (− 0.28 to − 0.02)	− 0.10 (− 0.24 to − 0.01)	0.130	0.897^b^
	rMTT	− 0.70 (− 1.22 to − 0.08)	− 0.19 (− 0.76 to 0.08)	2.260	0.024^b^
Basal ganglia	rCBF	0.16 (− 0.04 to 0.39)	− 0.10 (− 0.39 to 0.14)	2.913	0.004^b^
	rCBV	− 0.19 (− 0.38 to 0.06)	0.11 (− 0.38 to 0.17)	0.479	0.632^b^
	rTTP	− 0.07 (− 0.20 to 0.03)	− 0.01 (− 0.06 to 0.03)	2.179	0.029^b^
	rMTT	− 0.17 (− 0.60 to 0.03)	0.03 (− 0.23 to 0.17)	2.331	0.020^b^
Frontal lobe	rCBF	0.15 (0.06 to 0.30)	0.01 (− 0.25 to 0.19)	2.036	0.042^b^
	rCBV	− 0.23 (− 0.40 to 0.09)	− 0.24 (− 0.53 to 0.01)	0.667	0.505^b^
	rTTP	− 0.06 (− 0.13 to 0.05)	− 0.01 (− 0.09 to 0.06)	0.649	0.516^b^
	rMTT	− 0.16 (− 0.46 to 0.19)	0.01 (− 0.38 to 0.32)	1.579	0.114^b^

*rCBF* relative cerebral blood flow, *rCBV* relative cerebral blood volume, *rTTP* relative time to peak, *rMTT* relative mean transit time, *STA-MCA* superficial temporal artery-middle cerebral artery, *EDAS* encephaloduroarteriosynangiosis

^a^*t*-test

^b^Wilcoxon’s rank-sum test

## Discussion

At present, surgical revascularization is the primary treatment for MMD. Surgical revascularization is recommended for symptomatic patients to reduce cerebral ischemia and improve the neurological prognosis by increasing the intracranial blood flow [32, 33]. In this study, 84 patients underwent STA-MCA or EDAS, and the postoperative perfusion, clinical symptoms, and neurological functional status were significantly improved than preoperative; this finding is consistent with that of previous research [5, 7, 17]. In addition, the improvement of CTP after surgery was related to the change in clinical symptoms, which was consistent with previous studies [5, 21, 34].

However, the intergroup comparison showed that the changes in clinical symptoms in the STA-MCA group were significantly better than those in the EDAS group, which could be associated with different postoperative changes in cerebral perfusion between the two groups. The perfusion analysis showed that cerebral perfusion in the ipsilateral frontal lobe and basal ganglia was significantly improved postoperatively in the STA-MCA group but not in the EDAS group. The difference in perfusion improvement between the two groups might be associated with different surgical revascularization procedures. EDAS involves reactive revascularization through exposure, contact, and angiogenesis. This passive procedure may limit the area perfused by revascularization within the cerebral cortex and superficial cortical areas in the operative area without improving the hemodynamics of deep brain tissues and frontal lobes [21, 29, 35]. Conversely, the blood is directly supplied to the brain through the bypassed artery and promotes arterial network formation and arteriogenesis through pressure gradient and fluid shear force in STA-MCA anastomosis [36]. Theoretically, the direct revascularization procedure can change the pathological cerebral hemodynamic pattern of patients with MMD [12] not only by directly supplying blood to deep brain tissues (such as the basal ganglia) but also compensating for the blood supply to the ipsilateral frontal lobe through collateral circulation. In this way, STA-MCA anastomosis could better relieve such clinical symptoms secondary to cerebral ischemia as transient ischemic attack (TIA), leg weakness, and cognitive abnormalities. This finding supports the view of Choi et al. [21] that direct or combined revascularization should be considered for patients with MMD with impaired anterior cerebral artery territory perfusion. Furthermore, the degree of perfusion improvement in each brain area in the STA-MCA group was generally greater than that in the EDAS group, which might be another reason for better postoperative clinical symptoms.

In contrast to CTP and the changes in clinical symptoms results, the postoperative DSA-evaluated collateralization grades were not significantly different between the two groups. The relief of preoperative clinical symptoms is the result of improved cerebral circulation and metabolism, whereas the collateralization grade only utilizes vascular morphology to characterize blood supply, which cannot fully represent the hemodynamic status. Studies found that although some patients do not show evident collateral vessels on postoperative DSA, their hemodynamic parameters on perfusion images and clinical symptoms are significantly improved [5, 37], which might be associated with postoperative capillary angiogenesis and arteriole formation. Therefore, measurement of quantitative hemodynamics using CTP can be more effective in assessing surgical outcomes of different surgical procedures than imaging of vascular morphology using DSA.

The postoperative mRS scores were significantly lower than that of preoperative in both the STA-MCA and EDAS groups, but the postoperative mRS scores had no significant difference between the two groups of patients; it seemed to contradict the clinical symptoms. Reviewing the cases in this group, it was found that the relief of clinical symptoms in some patients was caused by reduced frequency of TIA, fatigue, and headache/dizziness, and the improvement of these clinical symptoms is not well considered by the mRS scores. Therefore, the two results are not contradictory and should be considered jointly in evaluating surgical outcomes for patients with MMD.

Our study had several limitations. Firstly, this was a single-center, retrospective analysis, with a relatively small sample size and observed indicators, and further research is needed for the reliability of the results. Secondly, the selection criteria for patient surgical methods are based on guidelines and the center’s experience; many patients undergoing EDAS were selected away from STA-MCA due to small STA caliber, which may also adversely impact the efficacy of the procedure. Furthermore, the study was not involved in the assessment of perfusion differences in the posterior cerebral artery territory, as this area is rarely affected in patients with MMD.

In summary, both STA-MCA anastomosis and EDAS are associated with good clinical outcomes in adult patients with MMD. CTP enables an objective assessment of postoperative cerebral hemodynamic changes in adult patients with MMD. Notably, the STA-MCA group showed a greater extent of postoperative perfusion improvement and a better degree of perfusion improvement compared to EDAS. These findings suggest that CTP can contribute to the understanding of differential changes in clinical symptoms between the two surgical revascularization procedures.

## Data Availability

The datasets used for analyses during the current study are available from the corresponding authors upon reasonable request.

## Abbreviations

CBF: Cerebral blood flow
CBV: Cerebral blood volume
CTP: Computed tomography perfusion
DSA: Digital subtraction angiography
EDAS: Encephaloduroarteriosynangiosis
MCA: Middle cerebral artery
mRS: Modified Rankin Scale
MTT: Mean transit time
STA-MCA: Superficial temporal artery-middle cerebral artery
TIA: Transient ischemic attack
TTP: Time to peak
